# *Candida auris* in Dog Ears

**DOI:** 10.3390/jof9070720

**Published:** 2023-06-30

**Authors:** Anamika Yadav, Yue Wang, Kusum Jain, Vijay Amrit Raj Panwar, Hardeep Kaur, Vikas Kasana, Jianping Xu, Anuradha Chowdhary

**Affiliations:** 1 Medical Mycology Unit, Department of Microbiology, Vallabhbhai Patel Chest Institute, University of Delhi, Delhi 110007, India; yadavanamika761995@gmail.com (A.Y.); kusumjain93@gmail.com (K.J.); vikaskasana532@gmail.com (V.K.); 2 Department of Zoology, Ramjas College, University of Delhi, Delhi 110007, India; hkaur53d@gmail.com; 3 Department of Biology, McMaster University, Hamilton, ON L8S 4K1, Canada; wangy660@mcmaster.ca (Y.W.); jpxu@mcmaster.ca (J.X.); 4 In-Patient Department, Sanjay Gandhi Animal Care Centre, New Delhi 110027, India; varspanwar@gmail.com

**Keywords:** otitis externa, meta-barcode sequencing, ITS, *Candida auris*, dogs, whole-genome sequencing

## Abstract

*Candida auris* is an emerging global public health threat and is resistant to most antifungal agents. Though fungi are significant pathogens for animals, the role of *C. auris* in animal health remains unexplored. Here, we analysed the microbial cultures of skin and ear swabs of 87 dogs in Delhi and performed fungal meta-barcode sequencing of ear and skin samples of 7 dogs with confirmed otitis externa (OE). Overall, 4.5% of dogs (4/87) with chronic skin infections contained evidence of *C. auris* in their ear canal (*n* = 3) and on their skin surface (*n* = 1). Of the three OE dogs with *C. auris* infection/colonisation, a diversity of fungi was observed, and their meta-barcode ITS sequence reads for *C. auris* ranged from 0.06% to 0.67%. Whole-genome sequencing of six *C. auris* strains obtained in culture from two dogs showed relatedness with Clade I clinical strains. The report highlights the isolation of *C. auris* from an animal source; however, the routes of transmission of this yeast to dogs and the clinical significance of transmission between dogs and humans remain to be investigated.

## 1. Introduction

*Candida auris* is a newly emerged human pathogenic yeast and is resistant to most antifungal agents. It has caused a diversity of infections and outbreaks in hospitals and presents a huge treatment challenge for clinicians and public health authorities. First reported in Japan in 2009, *C. auris* has spread rapidly worldwide [1,2,3] and caused the World Health Organisation to declare it as one of the four ‘critical priority’ fungal pathogens [4]. Outside hospital settings, *C. auris* has been isolated from the surface of stored apples, tidal marshes, hypersaline environments, and recently from wastewater, suggesting that this yeast can survive in harsh conditions [5,6,7,8]. Interestingly, trace sequences identical or highly similar to that of *C*. *auris* at the fungal barcode locus, i.e., the internal transcribed spacer (ITS) region, were found in metabarcoding data on an ear sample from a Spanish dog with otitis externa (OE) and from the skin of newts in the United Kingdom [9]. Though fungi are significant pathogens for animals, no live culture of *C. auris* has been isolated from animals, and its role in animal health remains to be explored [10]. We hereby report the isolation of *C. auris* from ear samples of two dogs. Additionally, culture-independent assessment of mycobiome analysis (DNA metabarcoding data) showed the presence of *C. auris*-specific ITS reads from the ear and skin of dogs with chronic skin infections.

## 2. Material and Methods

Swab collection and processing: In this study, we analysed the microbial communities of skin and ear swab samples in culture from a heterogeneous cohort of 87 dogs from a public referral hospital cum shelter in Delhi, India. The skin and ear swabs were collected and processed for bacterial and fungal cultures using the routine diagnostic protocol for suspected skin and ear infections. An ethics review was not requested by the Animal Care Centre because it was considered as a clinical observation study. The ear canal was sampled using sterile swabs (HIMEDIA, Mumbai, India) by rubbing the skin between the vertical–horizontal junctions. The skin swabs were obtained from the presenting lesions covering the dorsal–ventral skin, paws, and groin region. For microbiologic culture, the swabs were streaked directly on standard bacterial culture media, i.e., sheep blood agar (SBA) and MacConkey agar at 37 °C for 24 h. For fungal growth, Sabouraud dextrose agar (SDA) and CHROMagar (Becton, Dickinson, Baltimore, MD, USA) media were used and incubated at 37 °C for 24 h and 48 h, respectively. Additionally, for isolation of *Candida auris*, each swab was inoculated into yeast nitrogen base (YNB) broth with 10% NaCl at 37 °C for 4 days followed by streaking onto the SDA plates [7,11]. All microbial colonies, including bacteria and yeasts on blood agar, MacConkey agar, SDA, and CHROMagar, were identified by matrix-assisted laser desorption ionization–time of flight mass spectrometry (MALDI-TOF MS; Bruker Biotyper OC version 3.1, Daltonics, Bremen, Germany) with a score ≥ 2. *Candida auris* colonies obtained in culture were further confirmed by sequencing of internal transcribed spacer region (ITS) within the ribosomal RNA gene cluster [12].

Fungal DNA extraction and ITS amplicon sequencing (Metagenomics): In addition to samples for microbiological cultures, ITS meta-barcode sequencing was conducted for swab samples (skin, *n* = 2; ear swabs, *n* = 7) from seven dogs to study the fungal communities in these dogs with OE. Swabs were collected and placed in 0.05 M phosphate buffer for 30 min with a continuous shaking at 200 rpm. Swabs were discarded, and the solution was stored at −20 °C until subsequent analyses [7]. Microbial genomic DNA was extracted from each sample using a column-based QIAamp DNA minikit (Qiagen, Hilden, Germany) and quantified by QUBIT 4 fluorometer (ThermoFisher, USA) using DNA HS assay kit (ThermoFisher). Primers ITS-1, 5′-TCCGTAGGTGAACCTTGCGG-3′, ITS2, 5′-GCTGCGTTCTTCATCGATGA-3′, ITS3, 5′-GCATCGATGAAGAACGCAGC-3′, and ITS4, 5′-TCCTCCGCTTATTGATATGC-3 were used for amplifying the ITS1 and ITS2 regions, respectively. Illumina sequencing platform (NOVASEQ 6000, PIPITS v2.7) was used to perform ITS fungal sequencing, and PIPITS v2.7 was used for analysis [13]. Magic-BLAST was used to map the raw metabarcoding sequences of each sample against the fungal ITS database containing 15,695 fungal species [14]. The reference ITS sequences can be downloaded at https://ftp.ncbi.nlm.nih.gov/refseq/TargetedLoci/Fungi/. To calculate the relative read abundance at the species level in each sample, the read counts of each species were divided by the total reads mapping to the fungal ITS database. Then, the read abundance was pooled into the genera level, and the average read abundance of the samples for each genus was used to select the taxa for the heatmap. A total of 28 genera with average read abundance over 0.11% were included, and the 28 genera covered an average of 98% reads that were mapped to the database. A heatmap was generated using matplotlib and seaborn [15,16]. Additionally, in each sample, we specifically searched for ITS sequences of *C. auris*. For samples that contained *C. auris* ITS sequences (>94% sequence identity over the cleaned and filtered ITS reads), the relative frequencies of *C. auris* ITS sequences in each sample were calculated. To better reveal the differences among samples, the read abundance for each genus was standardized. The standardization was carried out for each taxon by subtracting the minimum and dividing each by its maximum.

Antifungal susceptibility testing: The susceptibility testing of yeasts was performed using the CLSI broth microdilution method, following M27-A3 [17]. The antifungals tested were fluconazole (FLU, Sigma, St. Louis, MO, USA), itraconazole (ITC, Lee Pharma, Hyderabad, India), voriconazole (VRC, Pfizer, Groton, CT, USA), posaconazole (POS, Merck, Whitehouse Station, NJ, USA), isavuconazole (ISA, Basilea Pharmaceutical, Basel, Switzerland), 5-flucytosine (5-FC, Sigma, St. Louis, MO, USA), micafungin (MFG, Astellas, Toyama, Japan), anidulafungin (AFG, Pfizer, Groton, CT, USA), and amphotericin B (AMB, Sigma, Groton, CT, USA). The drugs were tested for 10 (two-fold) dilutions, and the drug concentration ranges were as follows: FLU, 0.25–>128 mg/L; ITC, VRC, and AMB, 0.03–16 mg/L; POS, ISA, AFG, MFG, 0.015–8 mg/L; 5-FC, 0.125–64 mg/L [18]. *Candida krusei* strain ATCC6258 and *C. parapsilosis* strain ATCC22019 were used as quality controls. All statistical parameters were calculated using Prism version 6.00 (GraphPad Software).

Antibiotic susceptibility testing was conducted for all bacterial isolates using disk diffusion method following CLSI M100, Ed-32. For Gram-positive bacteria, including *Staphylococcus pseudointermedius*, *S. intermedius*, *S. simulans*, and *S. schleiferi*, eight drugs were tested, namely penicillin, ampicillin, clindamycin, vancomycin, azithromycin, linezolid, cefoxitin, and cotrimoxazole. For Gram-negative bacteria, i.e., *Acinetobacter baumanii*, and *Pseudomonas aeruginosa*, ciprofloxacin, levofloxacin, gentamicin, amikacin, meropenem, cefepime, ceftazidime, ceftazidime-clavulanate, piperacillin, and piperacillin-tazobactam were tested. The inhibitory zone diameters obtained around the antibiotic discs were measured after incubation for 24 h at 37 °C and evaluated [19].

Whole Genome Sequencing (WGS): Genomic DNA of all *C. auris* isolates (*n* = 6 obtained from ear swabs of two dogs with OE) were extracted using a column-based QIAamp DNA minikit (Qiagen, Hilden, Germany) and quantified by a QUBIT 3 Fluorometer using dS DNA HS Dye. WGS libraries were prepared using NEBNext ultra II DNA FS kit (New England Biolabs, Ipswich, MA, USA), as described previously, and sequenced on Illumina HiSeq 4000 [5,11]. For phylogenetic analysis to identify the clade affinity of our strains, one reference genome from each of the four major clades was included for comparison, including strains B8441 (clade I), B11220 (clade II), B11221 (clade III), and B11245 (clade IV). All strains were subjected to NASP pipeline for genome sequencing analysis, as described previously [7]. The phylogenetic tree was constructed using RAxML v8.0.25 under ASC_GTRCAT nucleotide substitution model and 1000 bootstrap replicates [20]. A maximum-likelihood tree was constructed based on SNPs present in at least one sample. This analysis revealed that all of our strains belonged to clade I (see Results below). We then proceeded to conduct an additional phylogenetic analysis using B8441 as reference and including 569 previously reported clade I *C. auris* samples from different countries, including Indian clinical strains [21], strains recovered from apples [7], and strains from the marine environment of the Andaman Islands [5]. In this broad clade I-specific analysis, SNP sites with ambiguous calls in over 0.5% of the samples were removed, and the remaining SNPs were concatenated for each sample. Maximum-likelihood phylogeny was constructed using RAxML-HPC2 on XSEDE in the CIPRES Science Gateway [22]. The tree uses the ASC_GTRCAT nucleotide substitution model and 1000 bootstrap iterations and was visualized with iTOL [23].

## 3. Results

Population description: Among 87 dogs, 52 (60%) were stray dogs who were admitted in the intensive care unit (ICU) with severe skin lesions due to chronic skin diseases (group IA and IB). In group IA dogs with skin infections, eight had otitis externa with clinical signs of ear infection, i.e., erythema, oedema, erosion, and exudate in the affected ear (Table 1). In group IB all of the remaining 44 dogs exhibited skin infections. The group II composed of 35 pet dogs (40%) who were attending the outpatient services for minor ailments of the gastrointestinal and urinary tracts.

Fungal and bacterial culture: Overall, the ear swabs of all 52 dogs (group IA and IB) yielded microbial growth. On the contrary, the skin samples of only 31% (*n* = 16) of the dogs had microbial growth, including the isolation of bacteria (*n* = 8) and yeast (*n* = 8) in eight dogs each. Furthermore, ear samples and skin samples of 14% (*n* = 7) and 4% (*n* = 2) of dogs contained both living yeasts and living bacteria. In the group IA and IB cohorts, *Candida tropicalis* (*n* = 3) and *Malassezia pachydermatis* (*n* = 3) were the predominant fungi on the skin (6% each), followed by *Candida glabrata* in a single dog. Also, in two (4%) dogs, *Acinetobacter baumanii* and *Staphylococcus pseudointermedius* were detected on the skin. In ear samples, *M. pachydermatis* was the predominant fungal species (*n* = 11, 21% dogs), followed by *C. tropicalis* (*n* = 8) in 15% cases (Table 1).

In group IA, all cases had confirmed ear infections, and the ear swabs yielded yeasts as the sole etiologic agent in four of eight cases. The ear swabs of the remaining four dogs had both yeasts and bacterial colonies in culture. Notably, a single dog with OE (D1 dog) showed colonisation with multiple yeasts in the ear skin, including *C. auris* (645-V-22), *Candida lusitaniae,* and *Candida rugosa*, along with *Acinetobacter baumanii*. The *C. auris*-positive dog (D1) was admitted with chronic skin infection and OE in the hospital for more than a month before our sampling, and the dog was on topical treatment with miconazole, mupirocin, and ofloxacin. A repeat sampling of the ear canal of this dog after four weeks again yielded *C. auris* (645-V-22_2). Furthermore, the ear swab obtained from another dog (D4) in group IB (with only skin infection) who had no symptoms of otitis was also positive for *C. auris*, yielding five colonies of *C. auris* (D_953_1 to D_953_5) in culture.

Interestingly, 63% (*n* = 22) of the group II cohort (*n* = 35) with no skin or ear infections showed microbial growth from ear and/or skin swabs, but none of the colonies were *C. auris*. Also, a high rate of skin colonisation (43%, *n* = 15) was noted in these otherwise healthy dogs, whereas ear samples of only seven dogs showed microbial growth. Of these seven dogs, both *Candida tropicalis* (*n* = 3) and *M. pachydermatis* (*n* = 3) were observed to be equally distributed in the ear samples, followed by *Candida krusei* (*n* = 2) and *Candida parapsilosis* (*n* = 1). The skin surface was predominately colonised by *C. tropicalis* (*n* = 3, 14%) *and C. lusitaniae* (*n* = 2, 9%), and bacterial colonisation was due to *A. baumanii* (*n* = 2, 5.7%) *and S. intermedius* (*n* = 2, 5.7%).

We investigated healthcare workers’ (HCW, *n* = 6) hand swabs for microbial growth. The ICU board of the Animal Facility guided the collection of hand swab cultures from healthcare workers (*n* = 6) handling the dogs and the environmental sampling of the ICU for microbial cultures. Also, the hand swab of a single HCW in the ICU was analysed for fungal diversity using metabarcoding. Hand colonisation by microbes was noted in five of the six HCWs. Overall, a varied spectrum of yeasts, including *C. krusei*, *C. parapsilosis*, *C. tropicalis*, and *Trichosporan asahii*, was observed on the skin surfaces of the hands of HCWs in culture. Additionally, the doors, benches, and floor of the ICU were found to be colonised by *C. tropicalis* and *C. krusei.*

**Figure 1:**
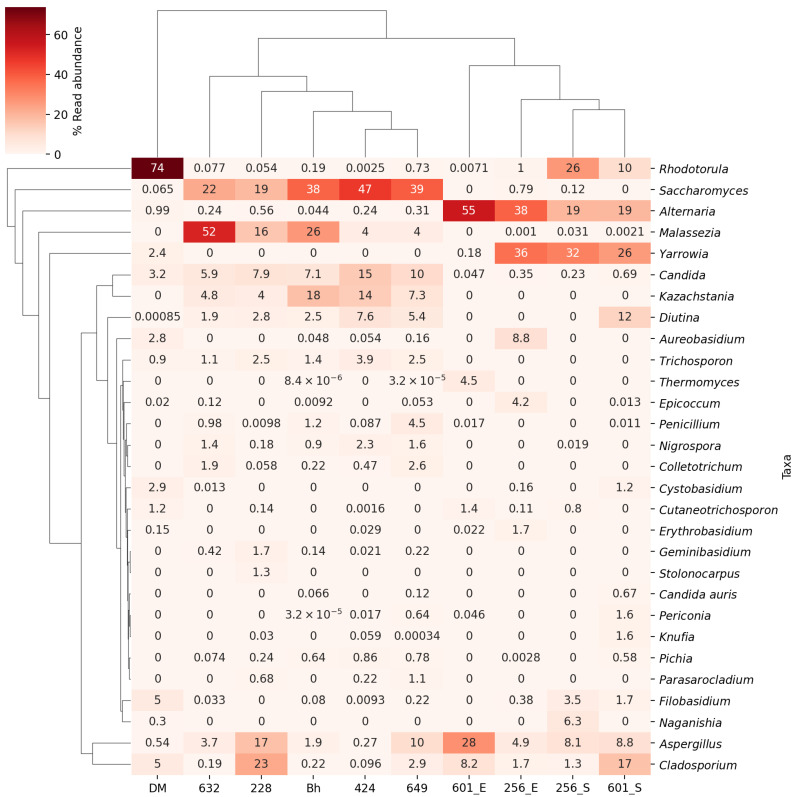
Heatmap showing the distribution of the 28 most common fungal genera in ear and skin samples from seven dogs and one healthcare worker. Different intensities of colours represent the percent read abundance of a genus in each sample. The dendrogram on the left is to indicate the similarity between different genera based on their read abundance profiles. Genera that are closer together on the dendrogram share more similar abundance patterns in our samples. The dendrogram on the top represents the clustering and relationships between the different samples based on their read abundance profiles of listed genera. Samples that are closer together on the dendrogram share more similar read abundance profiles.

Metagenomics analysis: According to metabarcoding data, evidence of *C. auris* was found in the ear skin samples of two dogs (both with OE) and on the skin surface of a single dog with OE (in the group IA cohort). A total of 248,515,598 ITS reads were obtained from ten samples (nine samples from dogs and one HCW’s hands), with an average of 24,851,560 reads per sample. After sequence alignment, 151,552,302 ITS reads were matched to a fungal ITS database with an average of 15,155,230 reads per sample. The length of the high-quality reads was 152 base pairs. The meta-barcode data from the ear of the dog with a positive culture for *C. auris* (D1) were also found positive for 34,394 ITS reads (0.12%) for *C. auris*. In addition, two dogs with negative culture for *C. auris* also showed the presence of 39,577 (0.06%) and 3231 (0.6%) ITS reads for *C. auris* from the ear (D2) and skin (D3), respectively. The heatmap shows the percentage of ITS read abundance of fungi in the 10 samples (Figure 1). The percent ITS reads for all fungal species are given in Table S1.

Interestingly, among the three dogs containing ITS sequences for *C. auris*, there seemed to be a negative correlation between the ITS read abundance for *Malassezia* species and that of *C. auris* (Table S1). In addition, a single ear sample with the highest percentage (52.2%) of ITS reads for *Malassezia* did not have traces of *C. auris*. However, there was a big variation in the frequencies of *Malassezia* ITS sequences (0–52.2%) among *C. auris*-negative dogs. Aside from *C. auris*, other *Candida* species were frequently recovered. Of the seven dogs with OE whose ear swabs were subjected to ITS amplicon sequencing, there was an average of 15,155,230 ITS reads per samples for *Candida* species. In addition, the presence of *C. tropicalis*, *C. krusei, C. parapsilosis* and *T. asahii*, in the microbial cultures of the hand swab of an HCW showed correlation with the presence of ITS reads (3.2%) of *Candida* species.

Antifungal susceptibility testing (AFST): The MIC data of the antifungals tested are summarised in Table S2. The two *C. auris* strains (645-V-22, 645-V-22_2) from the D1 dog, were found to be resistant to fluconazole (FLU) (MIC > 128 mg/L), while all of the other five strains isolated from the D4 case showed 2–4-fold lower MICs for FLU (32–64 mg/L). All *C. auris* strains were susceptible to the other tested drugs (Table S2).

All of tested isolates of *Staphylococcus*, *Pseudomonas*, and *Acinetobacter* genera were susceptible to all drugs except *S. pseudointermedius*, which was found to be resistant to clindamycin and azithromycin.

**Figure 2:**
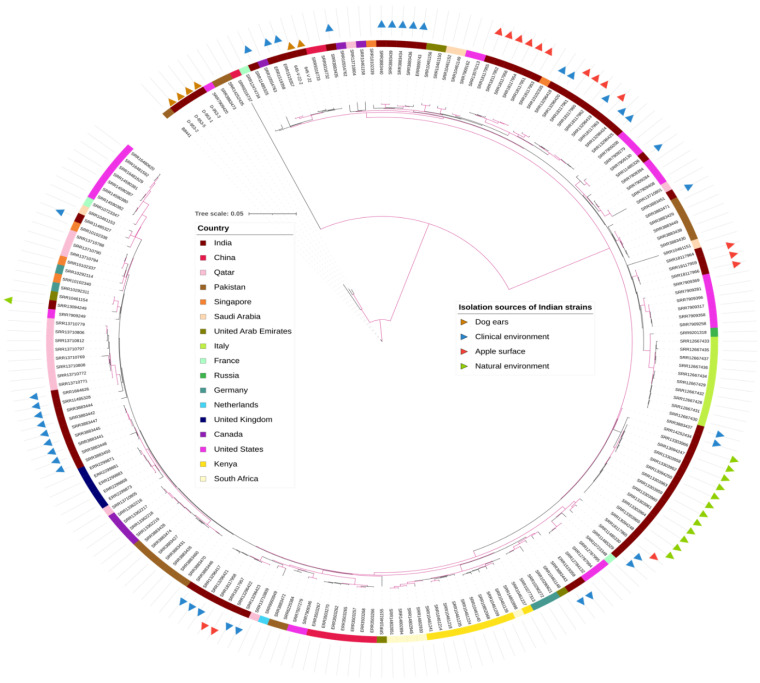
Genetic relationships of *C. auris* isolates from dog ears and clade I strains from other countries inferred based on concatenated genome-wide SNPs. Branches with bootstrap support over 0.95 are highlighted in purple. Indian strains were labelled with triangles in various colours to indicate their isolation sources. The inner colour strips specify the geographic location of the isolates.

Whole genome sequencing: WGS analysis revealed that two *C. auris* strains (645-V-22, 645-V-22_2) obtained from the D1 dog four weeks apart differed by four SNPs from each other, suggesting that the same strain persisted during the four weeks in the colonised ear. In the D4 dog, four of the five isolates were sequenced, and clonal clustering of all four strains of *C. auris* was observed with a 2–6 SNPs difference among the strains. Notably, these four strains (D_953_1, D_953_2, D_953_3, and D_953_5) showed a difference of 955–965 SNPs from *C. auris* strains isolated from the D1 dog, suggesting the circulation of genetically distinct *C. auris* strains. All four *C. auris* strains from the D4 dog clustered with B8441, a clade I reference strain (Pakistan). Interestingly, the *C. auris* strains from the D4 dog showed close genetic relatedness (8–11 SNPs difference) with the Pakistan clade I reference strain. Phylogenetic analysis of 569 global clade I *C. auris* strains grouped the two D1 strains with two *C. auris* strains causing candidemia (7–14 SNPs) in Delhi, India (Figure 2) [3,21]. These two strains from the D1 dog showed K143R amino acid substitution in the *ERG11* gene and A640V amino acid substitution in the *TAC1B* gene. Notably, four *C. auris* strains from the D4 dog showed the absence of mutation in azole-resistance-related genes, i.e., *ERG11* and *TAC1B.*

## 4. Discussion

Our report documents for the first time the isolation of live *C. auris* culture from an animal source. Overall, 4 of the 87 dogs (4.5%) contained evidence of *C. auris* infection or colonisation in their ear and on the surface of their skin. Notably, three of the four *C. auris*-positive dogs, either by culture (*n* = 2) or by ITS meta-barcode sequencing (*n* = 2), harboured this yeast in the ear canal. Furthermore, among the eight dogs in the OE cohort, two had *C. auris,* suggesting an association of *C. auris* with external ear canal infections.

*Candida auris* has been previously isolated from ear infections in humans from Korea, Japan, and Iran [1,24,25], but those strains mainly belonged to Clade II and Clade V [3,25,26]. In a recent study from Spain investigating the bacterial and fungal microbiota in the ear canals of dogs with OE, 12 (0.01%) *C. auris*-specific ITS reads out of a total of 81,213 ITS reads were found in a single dog [9,27]. In contrast, in the present study, an ample number of *C. auris* reads accounting for 0.06% and 0.12% was observed in the ear samples, much higher than that in the ear sample of the Spanish dog. In addition, the skin of a single dog was also found to be colonised by *C. auris*, with 3231 (0.67%) ITS reads. The genomic data of *C. auris* strains from the ears of two dogs showed that genetically distinct strains of Clade I were infecting or colonising the dogs. A close association of *C. auris* strains from a single dog (D4) with the Pakistan clade I reference strain suggested a clonal transmission of this yeast in the Indian subcontinent. Interestingly, this strain with a MIC value of 32 mg/L for fluconazole had no known mutations in genes related to azole resistance, i.e., in the *ERG11* and *TAC1B* genes. In contrast, *C. auris* strains from the D1 dog showed similarity with the Indian clinical strains and exhibited previously known K143R amino acid substitution in the *ERG11* gene and A640V amino acid substitution in the *TAC1B* gene, likely contributing to the high MIC of fluconazole (MIC > 128 mg/L).

It is important to emphasise that in the present study, *C. auris* was not detected on the healthy intact skin of pet dogs using the culture method. However, the ITS amplicon sequencing was not performed on the skin and ear swabs of otherwise healthy dogs. *C. auris* colonised the breached skin of the ears of dogs with chronic skin conditions admitted to the ICU, but colonisation of the fomites samples in the ICU was not observed. This scenario is unlike the colonisation by *C. auris* in humans, where the close inanimate environment is readily contaminated with this yeast due to shedding of skin scales. The fact that, in the present study, the skin of the ears of dogs was found to be colonised by *C. auris* rather than the exposed (ventral and dorsal) skin probably contributed to reduced shedding in the environment. It is worth emphasising the circulation of closely related *C. auris* clade I strain in humans and dogs. However, the prevalence of *C. auris* transmissions between humans and domesticated animals warrants further investigations. Finally, the association of *C. auris* with ears in humans and dogs hints toward the ear canal possibly being an important reservoir of this yeast.

## Figures and Tables

**Table 1:** Distribution of yeasts and bacteria on the skin and in the ear of 87 dogs collected from North Delhi, India.

Disease Categories in Dogs (Numbers)	Period of Sampling	Yeasts		Bacterial Isolation (Body Site, No. of Dogs Positive for the Given Species)
		Culture (Body Site, No. of Dogs Positive for the Given Species)	Amplicon Sequencing (ITS Reads for *C. auris*/Total Reads Matched to Fungal ITS; Percentage of ITS Reads)	
Group IA Chronic skin disease with otitis externa (*n* = 8),	27-09-22 to 17-04-23	*C. auris* (E *, *n* = 1) (D1) ^@^ *C. rugosa* (E, *n* = 2), *C. krusei* (E, *n* = 3) (D2) ^@^ *C. lusitaniae* (E, *n* = 1) *C. glabrata* (S ^#^, *n* = 1) (D3) ^@^	*C. auris* from ear swab of D1 dog (34394/27920633; 0.12%) *C. auris* from ear swab of D2 dog (39577/59853350; 0.06%) *C. auris* from skin swab of D3 dog (3231/479708; 0.67%)	*A. baumanii* (E, *n* = 1) *S. pseudointermedius* (S, *n* = 1)
Group IB Chronic skin disease (*n* = 44),	14-08-22 to 17-04-23	*C. auris* (E, *n* = 1) (D4) ^@^ *M. pachydermatis* (E, *n* = 11; S, *n* = 3) *C. tropicalis* (E, *n* = 8; S, *n* = 3), *T. asahii* (E, *n* = 7), *C. glabrata* (E, *n* = 6, S; *n* = 1), *C. rugosa* (E, *n* =1), *C. albicans* (E, *n* = 1),	ND	*A. baumanii* (E, *n* =15; S, *n* = 2) *S. pseudointermedius* (E, *n* = 9; S, *n* = 2) *S. intermedius*, (E, *n* = 1; S, *n* = 1) *K. pneumoniae* (E, *n* = 1) *S. simulans* (S, *n* = 1) *S. schleiferi* (S, *n* = 1) *P. aeruginosa* (E, *n* = 1)
Group II Negative for skin and ear infections (*n* = 35),	11-05-22 to 02-09-22	*C. tropicalis* (E, *n* = 3; S, *n* = 3) *M. pachydermatis* (E, *n* = 3; S, *n* = 1) *C. krusei* (E, *n* = 2) *C. lusitaniae* (S, *n* = 2) *C. parapsilosis* (E, *n* = 1)	ND	*A. baumanii* (E, *n* = 1; S, *n* = 2), *S. pseudointermedius* (E, *n* = 6), *P. aeruginosa* (E, *n* = 3; S, *n* = 1) *S. schleiferi* (E, *n* = 1; S, *n* = 1) *S. intermedius* (S, *n* = 2), *K. pneumoniae* (S, *n* = 1), *E. faecalis* (S, *n* = 1), *B. cereus* (E, *n* = 1)

* ear, ^@^ dogs positive for *C. auris* either by culture or ITS amplicon sequencing, ^#^ skin.

## Data Availability

Genomic and meta-barcode data of the present study are submitted under bioproject number PRJNA917187.

## Supplementary Materials

The following supporting information can be downloaded at: https://www.mdpi.com/article/10.3390/jof9070720/s1, Table S1: Distribution of the 28 most common fungal genera in the ten samples (9 samples from dogs and one HCW hands). Individual sheets list the percent ITS reads data for all fungal genera from ten samples. Table S2: In vitro antifungal susceptibility profile of 10 yeast species isolated from dogs (*n* = 87) against ten antifungal drugs using CLSI-BMD method.
