# Supplementary material for: Candida auris in Dog Ears
Source: J Fungi (Basel). 2023 Jun 30;9(7):720. doi: 10.3390/jof9070720 (PMC10381908; doi:10.3390/jof9070720)
Supplement: Supplementary file 1 [file jof-09-00720-s001.zip › Supplimentary file _22-06-23/Table S2.pdf]

Table S2. *In vitro* antifungal susceptibility profile of 10 yeast species isolated from dogs (n=87) against different antifungal drugs using CLSI-BMD method.

Species (no. of strains tested)	Parameters	MICs (mg/L)[a]								
		**FLU**	**ITC**	**VRC**	**ISA**	**POS**	**AMB**	**MFG**	**AFG**	**5-FC**
C. auris (n=7)	Range	32->128	0.06-1	0.03-0.5	0.12-0.25	0.015-0.5	0.25-0.5	0.25-1	0.01-0.06	0.125-0.5
	MIC_{50}[b]	64	0.06	0.03	0.125	0.015	0.5	0.5	0.03	0.125
	MIC_{90}[c]	>128	0.7	0.5	0.75	0.35	0.5	1	0.06	0.35
M. pachydermatis (n=15)	Range	1-4	0.03-0.5	0.03-0.06	0.01-0.12	0.25-0.5	0.5	1	0.5-1	0.06
	MIC_{50}	2	0.04	0.03	0.06	0.25	0.5	1	0.75	0.06
	MIC_{90}	3.4	0.36	0.05	0.10	0.42	0.5	1	1	0.06
C. tropicalis (n=17)	Range	1-2	0.06-0.5	0.03-0.25	0.01-0.12	0.06-05	0.03-2	0.03-0.12	0.01	0.06-0.25
	MIC_{50}	1	0.12	0.06	0.12	0.25	1	0.03	0.01	0.06
	MIC_{90}	2	0.5	0.06	0.12	0.45	2	0.06	0.01	0.22
C. krusei (n=8)	Range	16-32	0.25-1	0.12-0.5	0.03-0.5	0.5	0.03-1	0.12-0.5	0.01-0.12	1-16
	MIC_{50}	32	1	0.25	0.25	0.5	0.25	0.5	0.01	16
	MIC_{90}	32	1	0.35	0.35	0.5	0.7	0.5	0.08	16
T. asahii (n=7)	Range	0.25-1	0.03-0.06	0.03-0.06	0.01-0.012	0.25-0.5	1-8	2-8	8	2-8
	MIC_{50}	0.5	0.03	0.03	0.06	0.25	2.5	8	8	3
	MIC_{90}	0.75	0.06	0.045	0.09	0.37	8	8	8	6
C. lusitaniae (n=4)	Range	1-2	0.12-1	0.03-0.06	0.06-0.25	0.25	0.5-1	0.12-0.25	0.01-0.06	0.06-0.12

	MIC_{50}	1.5	0.56	0.04	0.15	0.25	0.75	0.18	0.03	0.09
	MIC_{90}	2	1	0.06	0.25	0.25	1	0.25	0.06	0.12
C. glabrata (n=6)	Range	2	0.5	0.5	1	1	0.5	1	0.5-1	0.06
	MIC_{50}	2	0.5	0.5	1	1	0.5	1	0.75	0.06
	MIC_{90}	2	0.5	0.5	1	1	0.5	1	1	0.06
C. rugosa (n=4)	Range	2	0.03-0.06	0.03-0.06	0.12-0.25	0.01-0.06	0.5-2	0.12-0.25	0.01-0.03	0.06-0.12
	MIC_{50}	2	0.03	0.03	0.25	0.01	0.5	0.25	0.03	0.06
	MIC_{90}	2	0.05	0.05	0.25	0.04	1.55	0.25	0.03	0.10
C. albicans (n=3)	MIC_{50}	2	0.03	0.06	0.25	0.5	0.25	0.25	0.03	0.5
C. parapsilosis (n=1)	MIC	1	0.125	0.06	0.06	0.25	1	0.5	0.5	0.06

[a]FLU, fluconazole; ITC, itraconazole; VRC, voriconazole; ISA, isavuconazole; POS, posaconazole; AMB, amphotericin B; MFG, micafungin; AFG, anidulafungin; 5FC, flucytosine. [b] MIC_{50}, MIC at which 50% of test isolates were inhibited. [C]MIC_{90}, MIC at which 90% of test isolates were inhibited.
